# Neural machine translation of clinical text: an empirical investigation into multilingual pre-trained language models and transfer-learning

**DOI:** 10.3389/fdgth.2024.1211564

**Published:** 2024-02-26

**Authors:** Lifeng Han, Serge Gladkoff, Gleb Erofeev, Irina Sorokina, Betty Galiano, Goran Nenadic

**Affiliations:** ^1^ Department of Computer Science, The University of Manchester, Manchester, United Kingom; ^2^ AI Lab, Logrus Global, Translation & Localization, Philadelphia, PA, United States; ^3^ Management Department, Ocean Translations, Rosario, Argentina

**Keywords:** Neural machine translation, clinical text translation, multilingual pre-trained language model, large language model, transfer learning, clinical knowledge transformation, Spanish-English translation

## Abstract

Clinical text and documents contain very rich information and knowledge in healthcare, and their processing using state-of-the-art language technology becomes very important for building intelligent systems for supporting healthcare and social good. This processing includes creating language understanding models and translating resources into other natural languages to share domain-specific cross-lingual knowledge. In this work, we conduct investigations on clinical text machine translation by examining multilingual neural network models using deep learning such as Transformer based structures. Furthermore, to address the language resource imbalance issue, we also carry out experiments using a transfer learning methodology based on massive multilingual pre-trained language models (MMPLMs). The experimental results on three sub-tasks including (1) clinical case (CC), (2) clinical terminology (CT), and (3) ontological concept (OC) show that our models achieved top-level performances in the ClinSpEn-2022 shared task on English-Spanish clinical domain data. Furthermore, our expert-based human evaluations demonstrate that the small-sized pre-trained language model (PLM) outperformed the other two extra-large language models by a large margin in the clinical domain fine-tuning, which finding was never reported in the field. Finally, the transfer learning method works well in our experimental setting using the WMT21fb model to accommodate a new language space Spanish that was not seen at the pre-training stage within WMT21fb itself, which deserves more exploitation for clinical knowledge transformation, e.g. to investigate into more languages. These research findings can shed some light on domain-specific machine translation development, especially in clinical and healthcare fields. Further research projects can be carried out based on our work to improve healthcare text analytics and knowledge transformation. Our data is openly available for research purposes at: https://github.com/HECTA-UoM/ClinicalNMT.

## 1 Introduction

Healthcare Text Analytics (HECTA) have gained more attention nowadays from researchers across different disciplines, due to their impact on clinical treatment, decision-making, hospital operation, and their recently empowered capabilities. These developments have much to do with the latest development of powerful language models (LMs), advanced machine-learning (ML) technologies, and increasingly available digital healthcare data from social media (1–3), and discharged outpatient letters from hospital settings (4–6).

Intelligent healthcare systems have been deployed in some hospitals to support clinicians’ diagnoses and decision-making regarding patients and problems (7, 8). Such usages include key information extraction (IE) from electronic health records (EHRs), normalisation to medical terminologies, knowledge graph (KG) construction, and relation extraction (RE) between symptoms (problems), diagnoses, treatments, and adverse drug events (9, 10). Some of these digital healthcare systems can also help patients self-diagnose in situations where no General Practitioners (GPs) and professional doctors are available (11, 12).

However, due to the language barriers and inequality of digital resources across languages, there is an urgent need for knowledge transformation, such as from one human language to another (13, 14). Thus, to help address digital health inequalities, machine translation (MT) technologies can be of good use in this case.

MT is one of the earliest artificial intelligence (AI) branches dating back to the 1950s, and it has gained a boom with other natural language processing (NLP) tasks in recent years due to the newly designed powerful learning model Transformers (15–18). Several attention mechanisms designed in Transformer deep neural models are proven to be capable of better learning from a large amount of available digital data compared to traditional statistical and neural network-based models (19–21).

**Figure 1:**
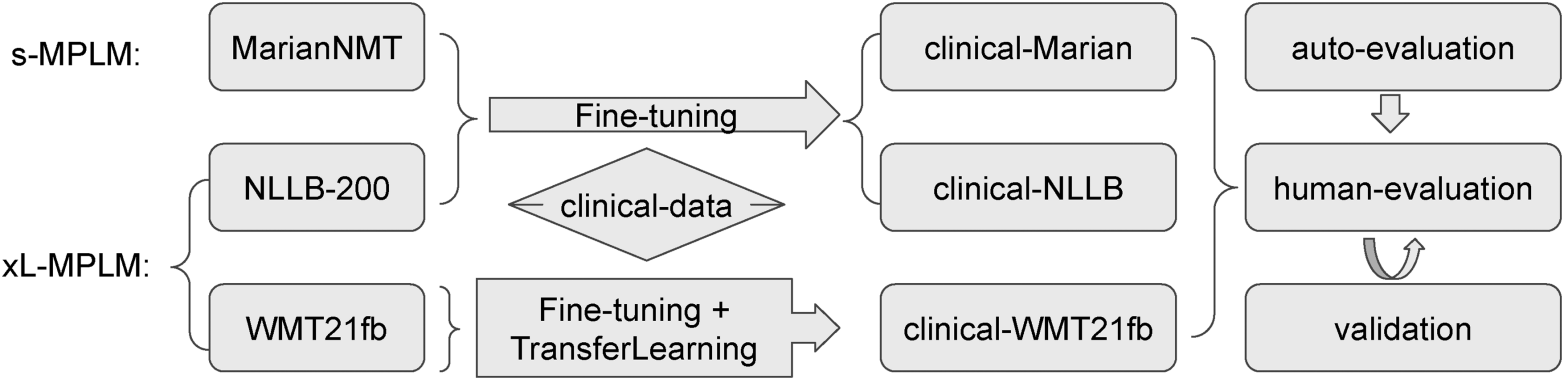
Illustration of the Investigation Workflow.

In this work, we investigate the state-of-the-art Transformer based Neural MT (NMT) models regarding clinical domain text translation, to facilitate digital healthcare and knowledge transformation with the workflow drawn in Figure 1. Being aware of some current development in the competition of language model sizes in NLP field, we set up the following base models for comparison study: (1) a small-sized multilingual pre-trained language model (s-MPLM) Marian, which was developed by researchers at the Adam Mickiewicz University in Poznan and at the NLP group in University of Edinburgh (22, 23); and (2) a massive-sized multilingual pre-trained LM (MMPLM/xL-MPLM) NLLB, developed by Meta-AI covering more than 200 languages (13). In addition to this, we set up a third model to investigate the possibility of transfer learning in the clinical domain MT: (3) the WMT21fb model which is another MMPLM from Meta-AI but with a limited amount of pre-trained language pairs including from English to Czech, German, Hausa, Icelandic, Japanese, Russian, and Chinese, and the opposite (24).

The testing language pairs of these translation models in our work are English ↔ Spanish. There aren’t other language pairs of openly available resources in the clinical domain MT as far as we know. We use the international shared task challenge data from ClinSpEn2022 “clinical domain Spanish-English MT 2022” for this purpose.^1^ ClinSpEn2022 was a sub-task of the BioMedical MT track at WMT2022 (25). There are three translation tasks inside ClinSpEn2022 including (i) clinical cases report; (ii) clinical terms, and (3) ontological concepts from the biomedical domain.

Regarding the evaluation of these LMs, we used the evaluation platform offered by ClinSpEn2022 shared task including several automatic metrics such as BLEU, METEOR, ROUGE, COMET. However, the automatic evaluation results did not give apparent differentiation between models on some tasks. Furthermore, there are issues like in-consistency regarding model ranking across automatic metrics. To address these issues and give a high-quality evaluation, we carried out an expert-based human evaluation step on three models using outputs of Task one “clinical case report”.

Our experimental investigation shows that (1) the extra-large MMPLM does not necessarily win the small-sized MPLM on clinical domain MT via fine-tuning; (2) our transfer-learning model works successfully for clinical domain MT task on language pairs that were not pre-trained upon but using fine-tuning. The first finding can shed some light on the research field that in clinical domain-specific MT, it is worthy to carry out more work on data cleaning and fine-tuning rather than building extra large LMs. Our second finding tells us the capability of MMPLMs in generating a new language pair knowledge space for translating clinical domain text even though this language pair was unseen in the pre-training stage with our experimental settings. This can be useful to low-resource NLP, such as the work by (26, 27).^2^

The rest of this article is organised as below: Section 2. surveys the related work to ours including clinical domain MT and NLP, large LMs, and transfer learning. Section 3. details the three LMs we deployed for comparison study. Section 4. introduces the experimental work we carried out and automatic evaluation outcomes. Section 5. follows up with expert-based human evaluation and the results. Finally, Section 6. concludes our work with discussion.

## 2 Related work

Applying NLP models to clinical healthcare has attracted much attention of many researchers, such as the work on disease status prediction using discharged summaries by Yang et al. (31), temporal expressions and events extraction from clinical narratives using combined methods of rules and machine learning by Kovačević et al. (32), using knowledge-based and data-driven methods for de-identification task in clinical narratives by Dehghan et al. (33), systematic reviews on clinical text mining and healthcare by Spasic et al. (5) and Elbattah and Dequen (34), etc.

However, using MT to help translate clinical text for knowledge transformation and help clinical decision-making is still a rising topic (14), even though it has been proven to be useful in the history for assisting *health communication* especially with post-editing strategies (35). This is partial because of the sensitive domain and high risk in clinical settings (36). Some of the recent progress on using MT for clinical text includes the work by Soto et al. (37) which leverages SNOMED-CT terms (38) and relations for MT between Basque and Spanish languages, Mujjiga et al. (39) which applies NMT model to identify semantic concepts in “abundant interchangeable words” in clinical domain and their experimental result shows NMT model can greatly improve the efficiency on extracting UMLS (40) concepts from a single document by using 30 milliseconds in comparison to traditional regular expression based methods which takes 3 seconds, and Finley et al. (41) which uses NMT to simplify the typical multi-stage workflow on clinical report dictation and even correct the errors from speech recognition.

With the prevalence of multilingual PLMs (MPLMs) developed from NLP fields, it becomes a current need to test their performances in the clinical domain of NMT. MPLMs have been adopted by many NLP tasks since the first emergence of the Transformer based learning structure (16). Among these, Marian is a small-sized MPLM led by Microsoft Translator based upon Nenatus NMT (42) with around 7.6 million parameters (22). Then, different research and development teams have been competing on the size of their LMs in recent years, e.g. the massive MPLMs (MMPLMs) WMT21fb and NLLB by Meta-AI which have the number of parameters set of 4.7 billion and 54 billion respectively (24, 13). To investigate the performances of these different models with varied model sizes towards clinical domain NMT with fine-tuning, we set up all these three models as our base models. To the best of our knowledge, our work is the first one regarding the comparison between small-size and extra-large MPLMs in the clinical domain of NMT.

Very close to the clinical domain, there has been a biomedical domain MT challenge series together held with the Annual Conference of MT (WMT), since 2016 (43, 44). The historical biomedical MT task covered a corpus of biomedical terminologies, scientific abstracts from Medline, summaries of proposals for animal experiments, etc. In 2022, it was the first time that this Biomedical-MT shared task introduced clinical domain data for Spanish-English language pairs (25).

As the WMT21fb model does not include Spanish in its pre-training, we also examine the transfer learning technology into the clinical domain NMT towards Spanish-English using the WMT21fb model. Transfer-learning (45) has proved useful for text classification and relation extraction (46, 47), and low-resource MT (48) fields. However, to the best of our knowledge, we are the first to test clinical domain NMT via transfer learning using MMPLMs.

## 3 Experimental designs

In this section, we introduce more details about the three MPLMs that we investigate in this work, i.e., Marian (22), WMT21fb (24), and NLLB (13).

### 3.1 Multilingual Marian NMT

**Figure 2:**
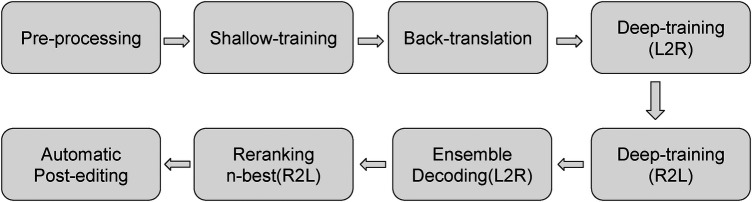
Marian Pre-Trained NMT - Training Pipeline.

Firstly, we draw a training diagram of the original Marian model on its pre-training steps in Figure 2 according to (22). The pre-processing step includes tokenisation, true-casing, and Byte-Pair Encoding (BPE) for sub-words. The shallow training is to learn a mid-phase translation model to produce temporary target outputs for back-translation. Then, the back-translation step produces the same amount of input source sentences to enlarge the corpus. The deep-training step first uses four left-to-right models which can be RNN (42) or Transformer (16) structures, which is followed by four right-to-left models in the opposite direction. The ensemble-decoding step will generate the n-best hypothesis translations for each source input segment, which will be re-ranked using a re-scoring mechanism. Finally, in Marian NMT, there is an automatic post-editing step integrated before producing the output. This step is also based on an end-to-end neural structure by modelling the *set*(MT-output, source sentence)→“post-edited output” as introduced by Junczys-Dowmunt and Grundkiewicz (49).

The Marian NMT model we deployed is from the Language Technology Research Group at the University of Helsinki led by Tiedemann and Thottingal (50) which is based on the original Marian model but continuously trained on the multilingual OPUS corpus (51) to make the model available to broader languages. It includes Spanish↔English (es↔en) pre-trained models and has 7.6 million parameters for fine-tuning.^3^

### 3.2 Extra-large multilingual WMT21fb and NLLB

**Figure 3:**
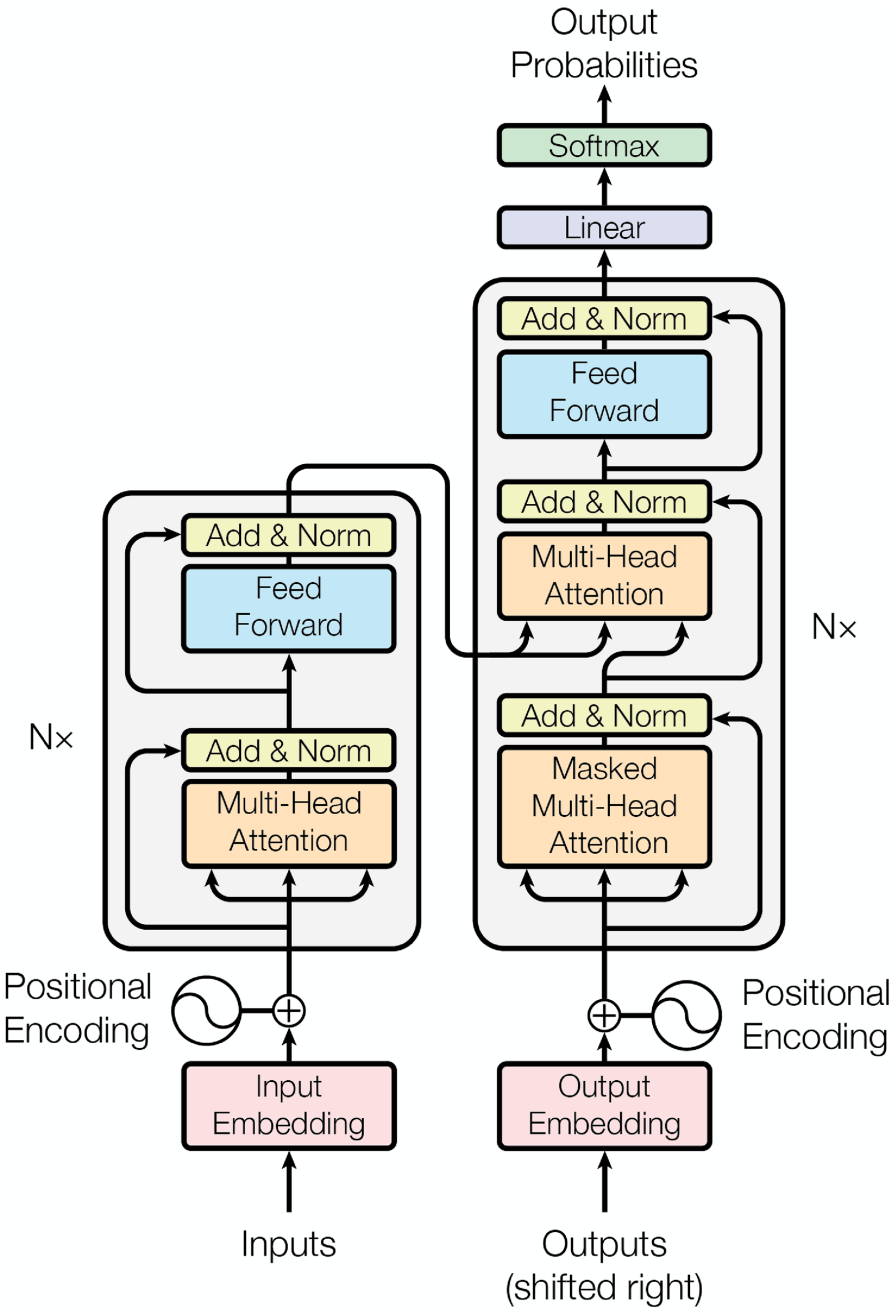
Several Attention based Transformer NMT structure (16).

Instead of the optional RNN structure used in the Marian model, both massive-sized multilingual PLMs (MMPLMs) WMT21fb and NLLB adopted Transformer as the main methodology. As shown in Figure 3, Transformer’s main components for encoder include position encoding, Multi-Head Attention, and Feed-Forward Network with layer normalisation at both two steps. The decoder uses Masked Multi-Head Attention to constrain the generation model only taking the already generated text into account.

**Figure 4:**
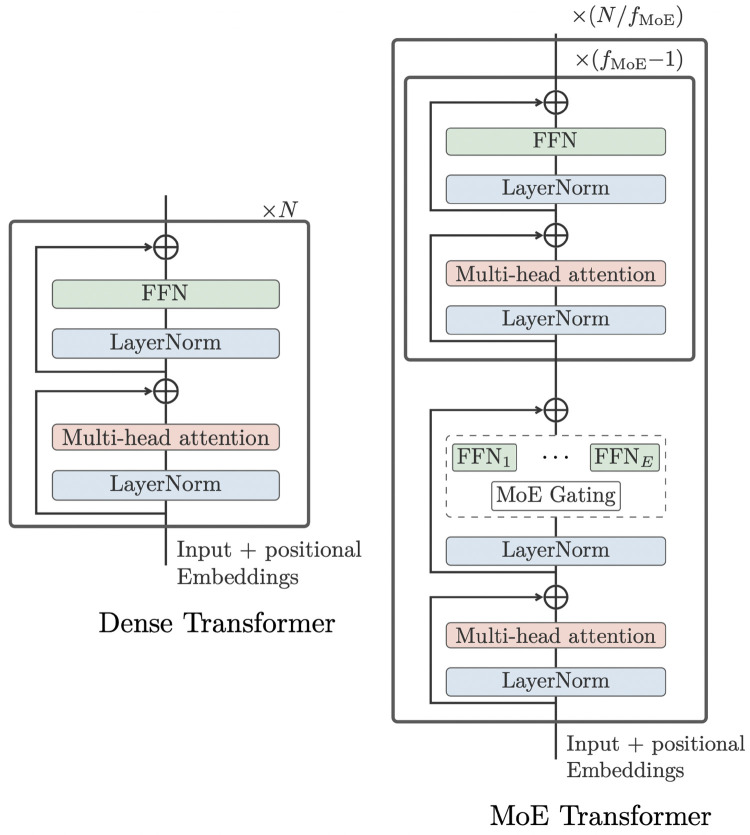
original dense Transformer (left) vs MoE Transformer (right) (13).

To increase the model capacity without making the extra-large model too slow for training, inspired by the work from Lepikhin et al. (52), the WMT21fb model included “Sparsely Gated Mixture-of- Expert (MoE)” models into the FFN layer of Transformer, as shown in Figure 4. The MoE model will only pass a sub-set of model parameters into the next level, thus decreasing the computational cost. However, this dropout is done in a random manner.

Furthermore, this structure design still needs language-specific training, such as English-to-other and other-to-English used by WMT21fb.

**Figure 5:**
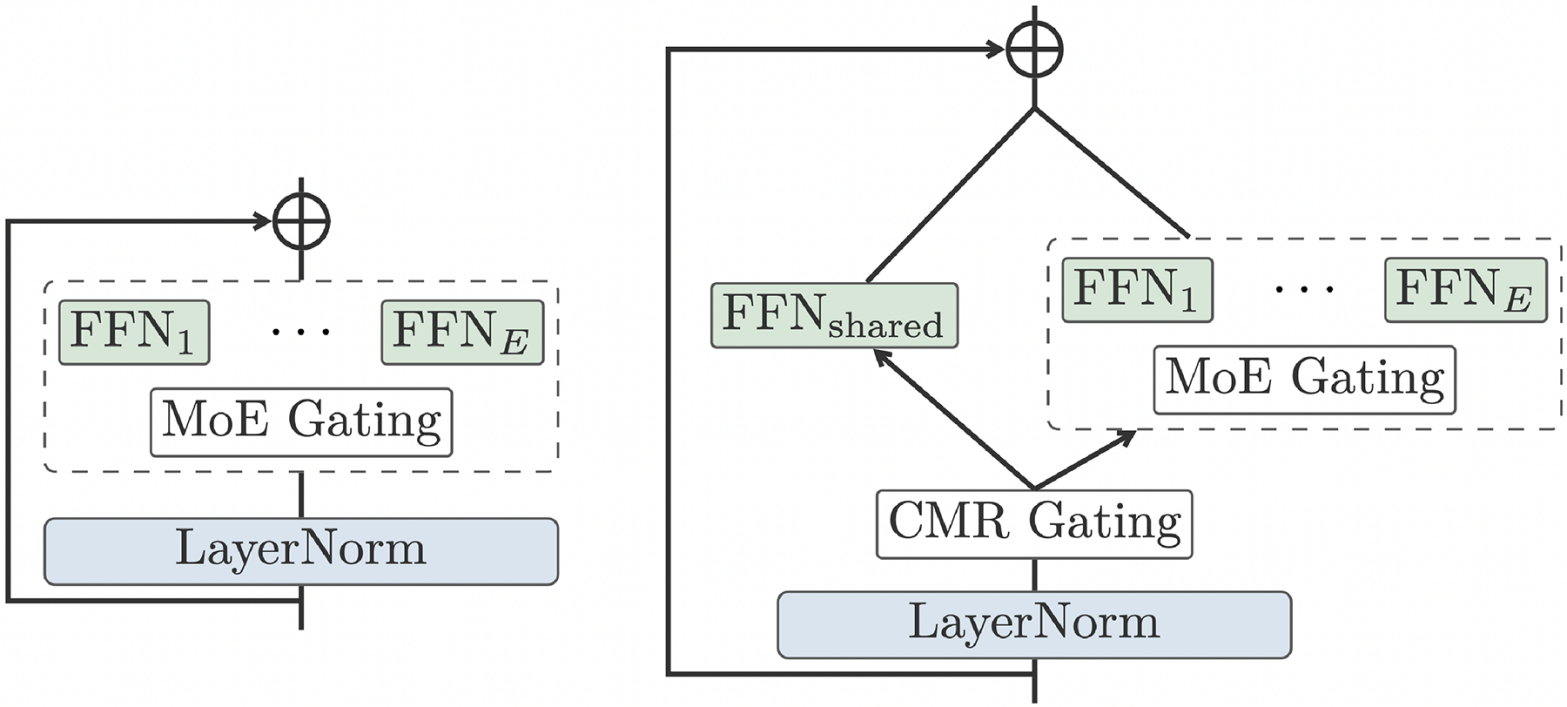
MoE vs Conditional MoE (13).

To further improve on this, the NLLB model designed a Conditional MoE Routing layer inspired by Zhang et al. (53) to ask the MoE model to decide which tokens to dropout according to their capacity demanding or routing efficiency. This is achieved by a binary gate, which assigns weights to dense FNN $FFN_{shared}$ or MoE Gating, as in Figure 5. The Conditional MoE also removes language-specific parameters for learning.

In summary, the WMT21fb and NLLB models share very similar learning structures, but most differently WMT21fb used language-specific constrained learning. The WMT21fb model we applied is ‘wmt21-dense-24-wide.En-X’ (and X-En direction) which has 4.7 billion parameters^4^ and contains the language pairs English ↔ Chinese, Czech, German, Hausa, Icelandic, Japanese, and Russian. The full NLLB model includes 200+ languages and has 54.5 billion parameters. Due to the computational restriction, we applied the distilled model of NLLB, i.e. NLLB-distilled, which has 1.3 billion parameters.

The WMT21fb model does not have Spanish in the trained language pairs, while NLLB includes Spanish as a high-resource language. This is a perfect setting for us to examine the transfer-learning technology on the clinical domain NMT by fine-tuning a translation model for the Spanish language on the WMT21fb model and comparing the output with the NLLB model (Spanish version).

## 4 Experimental settings and evaluations

### 4.1 Domain fine-tuning corpus

To fine-tune the three MPLMs for English ↔ Spanish language pair towards the clinical domain, we used the medical bilingual corpus MeSpEn from Villegas et al. (54), which contains sentences, glossaries, and terminologies. We carried out data cleaning and extracted around 250K pairs of segments on this language pair for domain fine-tuning of the three models. These extracted 250K pairs of segments are random from the original MeSpEn corpus and we divided them into a 9:1 ratio for training and development purposes. Because the WMT21fb pre-trained model did not include Spanish as one of the pre-trained language model, we could not use <2es> (to-Spanish) indicator for fine-tuning. As a solution, we used <2ru> as the indicator for this purpose (to-Spanish). This means a transfer learning challenge to investigate if the extra-large multilingual PLM (xL-PLM) WMT21fb has created a semantic space to accommodate a new language pair for translation modelling using the 250K size of corpus we extracted.

### 4.2 Model parameter settings

**Figure A1:**
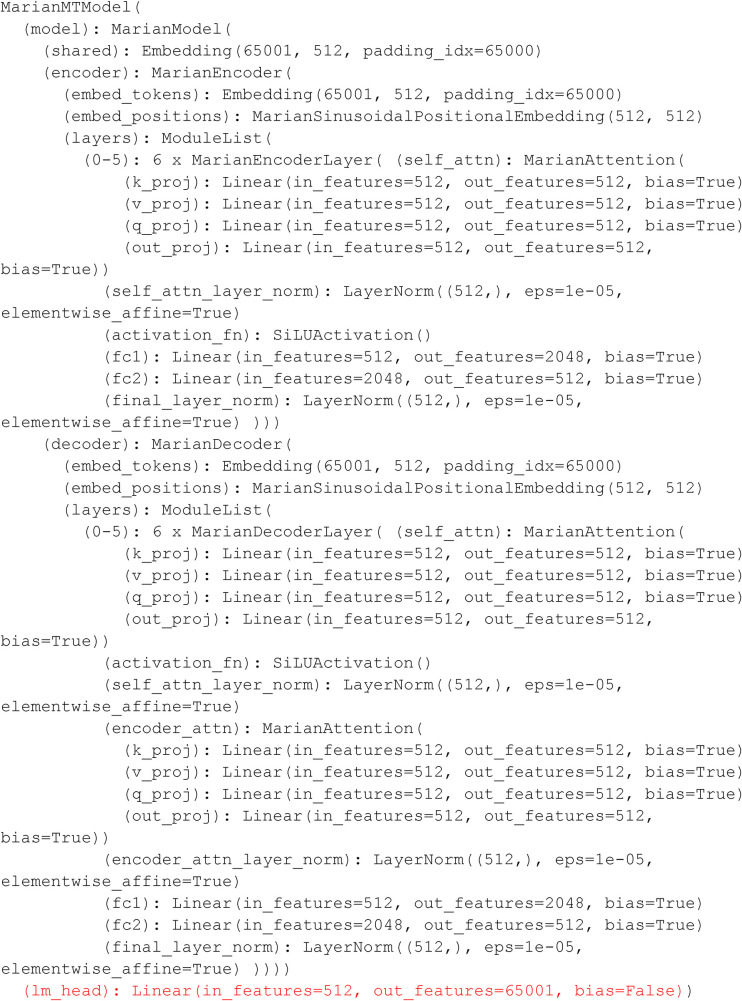
MarianNMT Fine-Tuning Parameters: Encoder and Decoder with 6+6 Layers.

Some parameter settings for s-MPLM Marian model fine-tuning are listed below. The last activation function for the generative model is a linear layer. Within the decoder and encoder, we used the Sigmoid Linear Units (SiLU) activation function. More detailed parameter and layer settings are displayed in Appendix Figure A1.

• learning rate =2e−5
• batch size =128
• weight decay −0.01
• training epochs =1
• encoder-decoder layers =6+6

Some fine-tuning parameters for NLLB-200-distilled (13) are listed below:

• batch size =24
• gradient accumulation steps =8
• weight decay =0.01
• learning rate =2e−5
• Activation function (encoder/decoder) = ReLU
• number of training epochs =1
• encoder-decoder layers =24+24

**Figure A2:**
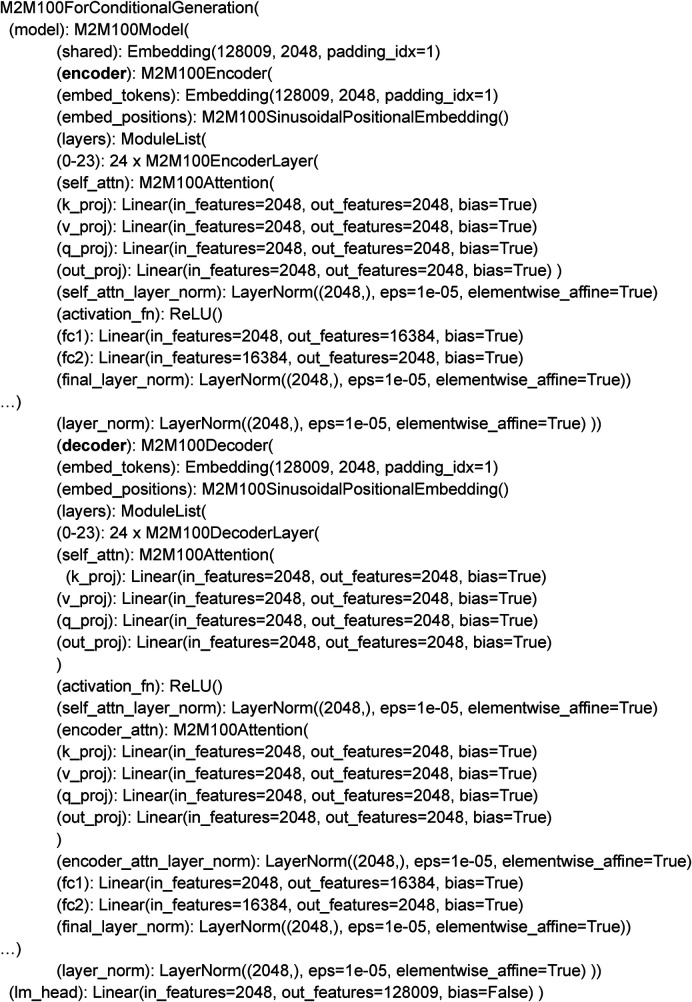
M2M-100 Model Structure For Conditional Generation: Encoder and Decoder Parameters with 24+24 Layers.

The fine-tuning parameters for WMT21fb model are the same as the NLLB-200-distilled, except for the batch size value which is set as 2. This is because the model is too large that we get out-of-memory (OOM) errors if we increase the batch size larger than 2. More details on M2M-100 parameters and layer settings for Conditional Generation Structure (55) we used for xL-MPLM WMT21fb and NLLB-200 can be found in Appendix Figure A2.

### 4.3 Test sets and automatic evaluations

The evaluation corpus we used is from the ClinSpEn-2022 shared task challenge data organised as part of the Biomedical MT track in WMT2022 (25). It has three sub-tasks: (1) EN→ES translation of 202 COVID19 clinical case reports; (2) ES→EN 19K clinical terms translation from biomedical literature and EHRs; and (3) EN→ES 2K ontological concept from biomedical ontology.

The automatic evaluation metrics used for testing include BLEU (HuggingFace) (56), ROUGE-L-F1 (57), METEOR (58), SacreBLEU (59), and COMET (60), hosted by the ClinSpEn-2022 platform.^5^ The metric scores are reported in Table 1 for three translation tasks. In the table, the parameter ‘plm.es’ is a question mark asking if the Spanish language was already included in the original off-the-shelf PLMs. For this question, both Marian and NLLB have Spanish in their PLMs, while WMT21fb does not, which indicates that Clinical-WMT21fb is a transfer learning model for EN↔ES language pair.

**Table 1:** Automatic Evaluation of Three MPLMs using ClinSpEn-2022 Platform. ‘plm.es’ means if the Spanish language is included in PLMs.

MT fine-tuning	plm.es	SacreBLEU	METEOR	COMET	BLEU	ROUGE-L-F1
Task-I: Clinical Cases (CC) EN→ES						
Clinical-Marian	Yes	*38.18*	*0.6338*	*0.4237*	*0.3650*	*0.6271*
Clnical-NLLB	Yes	37.74	0.6273	0.4081	0.3601	0.6193
Clinical-WMT21fb	No	34.30	0.5868	0.3448	0.3266	0.5927
Task-II: Clinical Terms (CT) EN←ES						
Clinical-Marian	Yes	26.87	*0.5885*	0.9791	0.2667	*0.6720*
Clinical-NLLB	Yes	*28.57*	0.5873	*1.0290*	*0.2844*	0.6710
Clinical-WMT21fb	No	24.39	0.5840	0.8584	0.2431	0.6699
Task-III: Ontology Concept (OC) EN→ES						
Clinical-Marian	Yes	39.10	*0.6262*	0.9495	0.3675	*0.7688*
Clinical-NLLB	Yes	*41.63*	0.6072	0.9180	*0.3932*	0.7477
Clinical-WMT21fb	No	40.71	0.5686	*0.9908*	0.3859	0.7199

From this automatic evaluation result, firstly, it is surprising that the much smaller-sized Clinical-Marian model won most of the scores across three tasks, indicated by *italic* font. Secondly, for two xL-MPLMs, even though the transfer-learning model Clinical-WMT21fb has a certain score gap to Clinical-NLLB on Task-1, it almost catches up with Clinical-NLLB for Task-2 and 3 even winning one score, the COMET for Task-3 (0.9908 vs 0.9180). This means the xL-MPLM has the capacity to create a multilingual semantic space and the capability to generate a new language model as long as there is enough fine-tuning of the corpus for this new language. Thirdly, there are issues with automatic metrics. This includes the confidence level on score difference (significance test), such as the very closely related scores for Task-1 on the first two winner models. In addition, the winner models change across Task-2 and 3 via different metrics.

We also observed that there are 4 percent of Russian tokens in the EN → ES output from Clinical-WMT21fb model. This indicates that the model keeps Russian tokens when it does not know how to translate the English token into Spanish. This is very interesting since the Russian tokens reserved in the text are not-nonsense, instead, they are meaning correct tokens, just in a foreign language. This might be the reason why COMET generated higher score for Clinical-WMT21fb model than Clinical-NLLB on Task-3 ‘ontological concept’, since COMET is a neural metric that calculates the semantic similarity on an embedding space, ignoring the word surface form.

To improve the trustworthiness of our empirical investigation and generate more clear evaluation output across three models, we carry out human expert-based evaluations in the next section.

### 4.4 Comparisons

To compare our much smaller sized clinical-Marian model with other existing work on this shared task data, such as Optum (61) and Huawei (62), we list the automatic evaluation scores in Table 2 where Optum attended all three sub-tasks, while Huawei only attended Task II: Clinical Terminology (CT). From the comparison scores using automatic metrics, we can see that much smaller-sized Clinical-Marian wins some metrics in each of the tasks. In addition, Optum used their in-house clinical data as extra training resources in addition to WMT offered training set, while the 250K training set we used for Clinical-Marian is extracted only using WMT data. Huawei’s model only wins one metric (COMET) out of five metrics on Task-2 (CT), however, both Clinical-Marian and Optum wins two metrics out of five. So there is not much better performance from Huawei on this task even though they have much more online resources and computational support.

**Table 2:** Model Comparisons on 3 Tasks between Clinical-Marian and Others.

Models	SacreBLEU	METEOR	COMET	BLEU	ROUGE
Task-1: Translating Clinical Cases					
Clinical-Marian	38.17	0.6337	0.4237	0.3650	0.6270
Optum	38.12	0.6447	0.4425	0.3642	0.6285
Task-2: Clinical Terminologies					
Optum	44.97	0.5880	1.1197	0.4396	0.7479
Huawei	41.57	0.624	1.190	0.4132	0.721
Clinical-Marian	39.10	0.6261	0.9494	0.3674	0.7688
Task-3: Translating Ontology Concepts					
Optum	44.97	0.5880	1.1197	0.4396	0.7479
Clinical-Marian	39.10	0.6261	0.9494	0.3674	0.7688

## 5 Human evaluation

As observed in the last section, there are two motivations for us to set up the expert-based human evaluation: (1) it is really surprising that the much smaller-sized MPLM (s-MPLM) Clinical-Marian wins the xL-MPLMs Clinical-NLLB and Clinical-WMT21fb; (2) to verify the hypothesis from automatic evaluation that Clinical-Marian really performs the best.

### 5.1 Human evaluation setup

To achieve both qualitative and quantitative human evaluation, we deployed a human-centric expert-based post-editing quality evaluation metric called HOPE by Gladkoff and Han (63) (it is also called LOGIPEM invented from Logrus Global LLC, a language service provider). HOPE evaluation metric has 8 predefined error types and each error type has corresponding different levels of penalty points according to the severity level. The sentence level and system level HOPE score is a comprehensive score reflecting the overall quality of outputs.

Firstly, we recruited 5 human evaluators who have backgrounds as professional translators, linguists, and biomedical researchers. For the evaluation data set, we take all the test set output from Task-1 ‘clinical case’ reports since this is the only task with full sentences. For the other two tasks on term and ontology level translation, MT engines can perform relatively good outcomes even without an effective encoder-decoder neural model, e.g. via a well prepared bilingual dictionary. We prepared 100 strings for each set and delivered all the sets to 5 professional evaluators.^6^ The tasks consisted of strings of medical cases going in order one by one, so the context of each case was clear to the evaluator.

Firstly, each one of them was given three files for evaluation from different engines, and instructions were given on both the online Perfectionist tool to be used for evaluation and the HOPE metrics. To ensure the human evaluation quality, we have also asked the strictest reviewer/evaluator to validate the work from other evaluators. The strictest reviewer is one of our specialists from the language service provider industry and has our trust according to their long term experiences in post-editing MT outputs and selecting MT engines in real world projects. The strictest reviewer gave better distinctions among all three evaluated models, while the less-strict reviewers sometimes gave similar scores to these models without picking their errors strictly.

### 5.2 Human evaluation output

**Figure 6:**
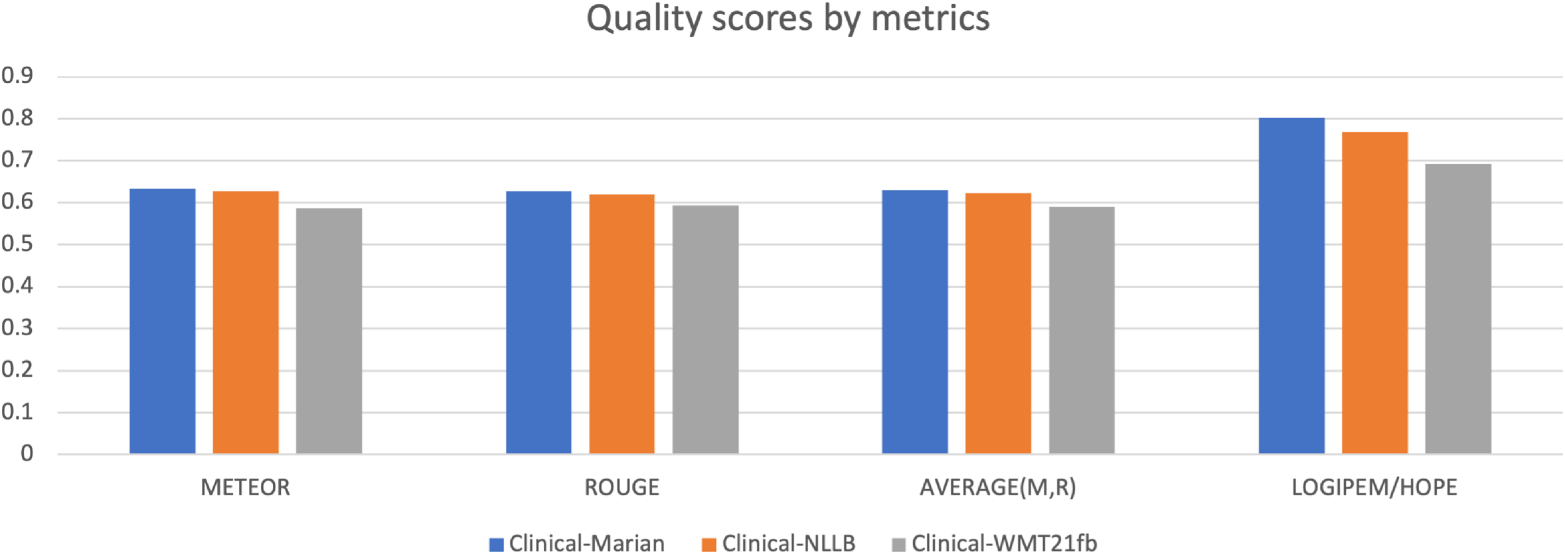
Comparison of Automatic Evaluations against Human Evaluation (HOPE).

The results of the evaluation can be seen in the online Perfectionist tool used, as downloaded from the tool in the form of the familiar Excel scorecards. They are tallied in Figure 6 and Table 3. The human evaluation result clearly shows which model is the best with large score gap in-between, i.e. the Clinical-Marian with score 0.801625, followed by Clinical-NLLB and Clinical-WMT21fb with scores 0.768125 and 0.692429 respectively.

**Table 3:** Automatic Evaluations vs Human Evaluations (HOPE) on Three MPLMs

MPLMs	Auto. Metrics		Average		Diff. in scores	
	METEOR	ROUGE	Averge(M,R)	HOPE	Auto.	HOPE
Clinical-Marian	0.6338	0.6271	0.6304	0.8016	6.45%	13.62%
Clnical-NLLB	0.6273	0.6193	0.6233	0.7681	1.13%	4.18%
Clinical-WMT21fb	0.5868	0.5927	0.5898	0.6924	5.38%	9.85%

To compare the human evaluation outputs with the automatic metric scores, we also added two metrics into the figure, i.e. METEOR and ROUGE, and the average score of these two metrics. The reason we choose these two is that they have a relatively positive correlation to human judgements. For the other three metrics, firstly, BLEU shows NLLB as better for terms and concepts, which does not correspond to human judgement. More than that, BLEU shows WMT21fb concepts to be better than the Marian Helsinki model, which is completely incorrect. Secondly, COMET score for the NLLB model is higher than 1, which is clearly caused by the fact that this implementation of COMET was not normalised by the Sigmoid function. Also, this COMET score for NLLB is higher than the one for Marian Helsinki. Another error is that the COMET score for clinical cases is much better than for both Marian and NLLB, which is completely impossible due to the presence of foreign language tokens in WMT21fb output. Finally, when we see COMET scores like 0.99 and 0.949 for Concepts, the score 0.42, 0.40 and 0.34 for Cases look evidently out of whack. BLEU-HF scores for all content types are ridiculously low on the scale of [0, 1] for both Cases and especially for Terms.

We have some findings from the comparisons.

• Most importantly, all human evaluators consistently showed positive correlation with preliminary human judgement of the MT output quality. Some of them were stricter than the others, but all of them rated the worst model as the worst and the best model as the best with only one exception. Results of human evaluation fully confirm the initial hypothesis about the quality of outputs of different engines, which is based on initial holistic spot-check human evaluation.
• LOGIPEM/HOPE metric shows the difference in the quality much bigger than any of the automated metrics. Where the automatic score shows 6 percent difference, human evaluation gives 14 percent. In other words, human linguists see the significant difference between the output quality of different engines very clearly. Even less trained evaluators show a positive correlation with the hypothesis.
• Even for those automatic metrics which correlate with human judgement, the score values do not seem to be representations of the uniform interval of [0, 1]. LOGIPEM/HOPE score will be exactly 1 if the segments, in reviewer’s opinion, do not have to be edited, and LOGIPEM/HOPE score 0.8 means only about 20% of work left to be done on the text with that score, since LOGIPEM/HOPE scoring model is designed with productivity assumptions in mind for various degrees of quality. COMET or ROUGE score 0.6 means that MT generated words different from those in the reference, this means that a perfect translation which is different from the reference would be rated much lower than 1. This is a huge distortion of linearity, which is metric-specific because all scores for different metrics live in their own ranges. Automatic scores appear to live on some sort of non-uniform scale of their own, which is yet another reason why they are not comparable to each other. The scale is compressed, and the difference between samples becomes statistically insignificant.
• The margin of error for all three engines is about 6%, which is about the same as the difference between mean of the measurements for different engines. This means that the difference between measurement is statistically significant, but a lot depends on the subjectivity of the reviewer, and the difference between reviewers’ positions may negate the difference in scores. However, even despite the subjectivity of the reviewers, groups of measurements for different engines appear to provide the statistically and visually significant difference.
• In general, human evaluators are to be trained / highly experienced, and need to maintain a certain level of rigour. The desired target quality should be stipulated quite clearly by customer specifications, as defined in ISO 11669 and ASTM F2575. To avoid incorrect (inflated) scores and decrease Inter-Rater Reliability (IRR), linguists must be tested prior to doing evaluations, or cross-validated.
• One evaluation task only takes 1 hour. There were 24 evaluation tasks in total, each task with 100 segments. It does not require setting up any data processing, software development, reference “golden standard” data or model-trained evaluation metric, it is clearly faster, more economical and reliable than research on whether automatic metric even pass the positive correlation test with human judgement (3 out of 5 did not in our case). While individual human measurements have variance, they are all valid, all correlate with human judgement if done with minimal training and rigour.
• Automatic metrics cannot be comparable across different engines, different data sets, different languages and different domains. On the contrary, human measurement is a golden universal ruler which provides the least common denominator between these scenarios. In other words, if Rouge is 0.67 for En-Fr for medical text, and Rouge is 0.82 for En-De for automotive text, we can’t compare these numbers. In contrast, LOGIPEM/HOPE score would mean one and the same thing across the board.

All of the above confirms the validity and interoperability of our human evaluation using LOGIPEM/HOPE metrics, which can be used as a single quick and easy validator of automatic metrics, ultimate fast and easy way to carry out analytic quality measurement to compare the engines and evaluate the quality of translation and post-editing.

### 5.3 Inter-rater-reliability

**Figure 7:**
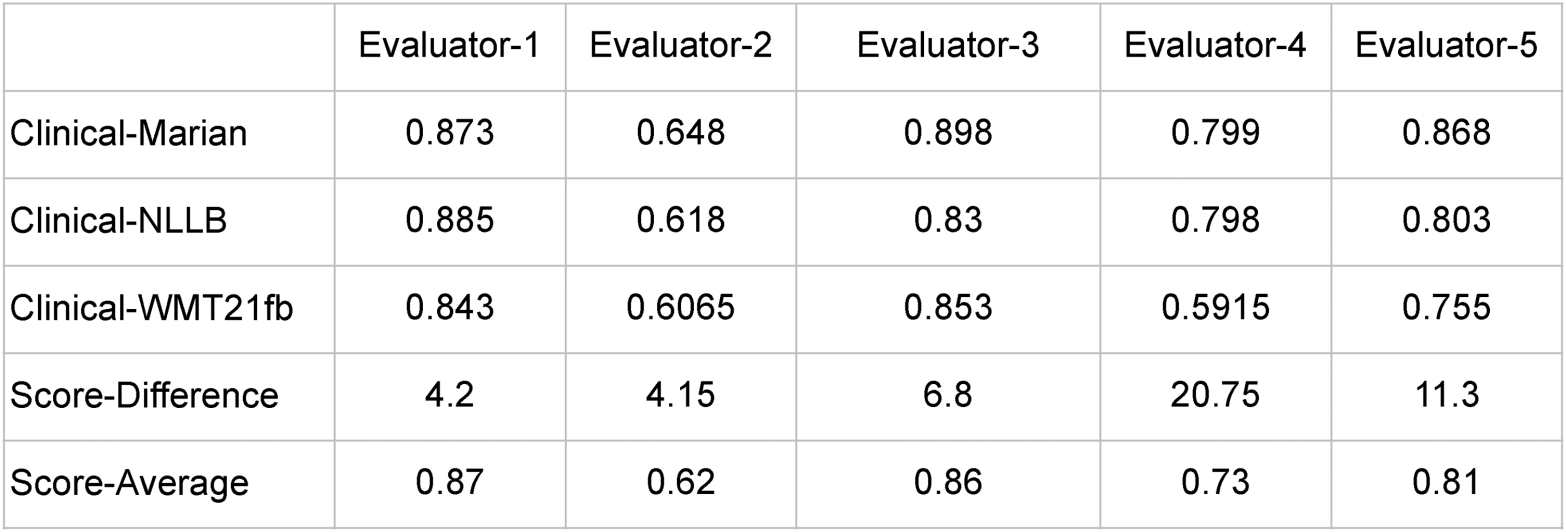
Summary of Human Expert-Based Evaluations.

To measure the inter-rater-reliability (IRR) of the human evaluation we carried out, in Figure 7, we summarise the evaluation output from five human evaluators on three models. The summaries include the average scores for each model, the score difference between these three models, and the average scores from the three models, from each person.

In this case we have continuous ratings (ranging from 0 to 1) rather than categorical ratings. Therefore, Cohen’s Kappa or Fleiss’ Kappa are not the most appropriate measures for this work. The Intraclass Correlation Coefficient (ICC) which measures the reliability of ratings by comparing the variability of different ratings of the same subject to the total variation across all ratings and all subjects would also not be appropriate here because there is a greater variation within the ratings of the same MT engine than between different MT engines.

However, we can compute standard deviations of the evaluations by different reviewers for each engine as follows:

• Marian: approximately 0.101
• NLLB: approximately 0.100
• WMT21: approximately 0.125

These values represent the amount of variability in the ratings given by different reviewers for each engine. The confidence intervals for these measurements for confidence level 80% are:

• Marian: approximately (0.759, 0.875)
• NLLB: approximately (0.729, 0.844)
• WMT21: approximately (0.658, 0.802)

In other words, with 80% confidence:

• Marian: 0.817 ± 0.058
• NLLB: 0.7865 ± 0.0575
• WMT21: 0.73 ± 0.072

**Figure 8:**
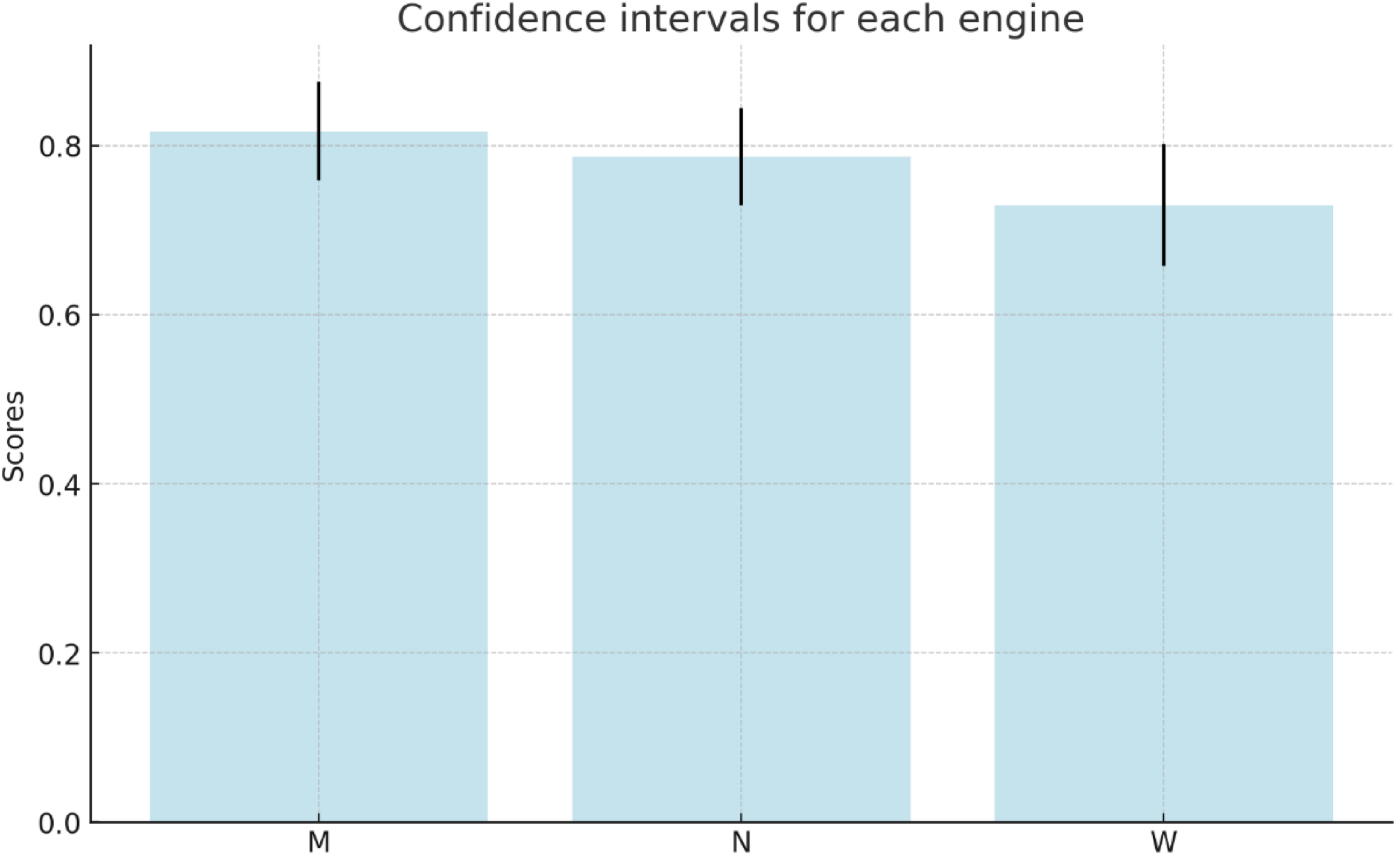
Confidence Intervals of Three Models (M, N, W): Clinical-Marian, Clinical-NLLB, and Clinical-WMT21fb.

This can be visualised in Figure 8. These intervals indeed overlap; however, Marian is reliably better than NLLB, and it is of course extremely surprising that WMT21fb rating is that high, considering that this result has been achieved with transfer learning by fine-tuning the engine without English-Spanish in the original PLM training dataset! As we can see, for some reviewers who are quite tolerant to errors (e.g. Evaluator-1) the quality of all the engines is almost the same. The more proficient and knowledgeable the reviewer is, the higher is the difference in their ratings.

### 5.4 Error analysis

**Figure 9:**
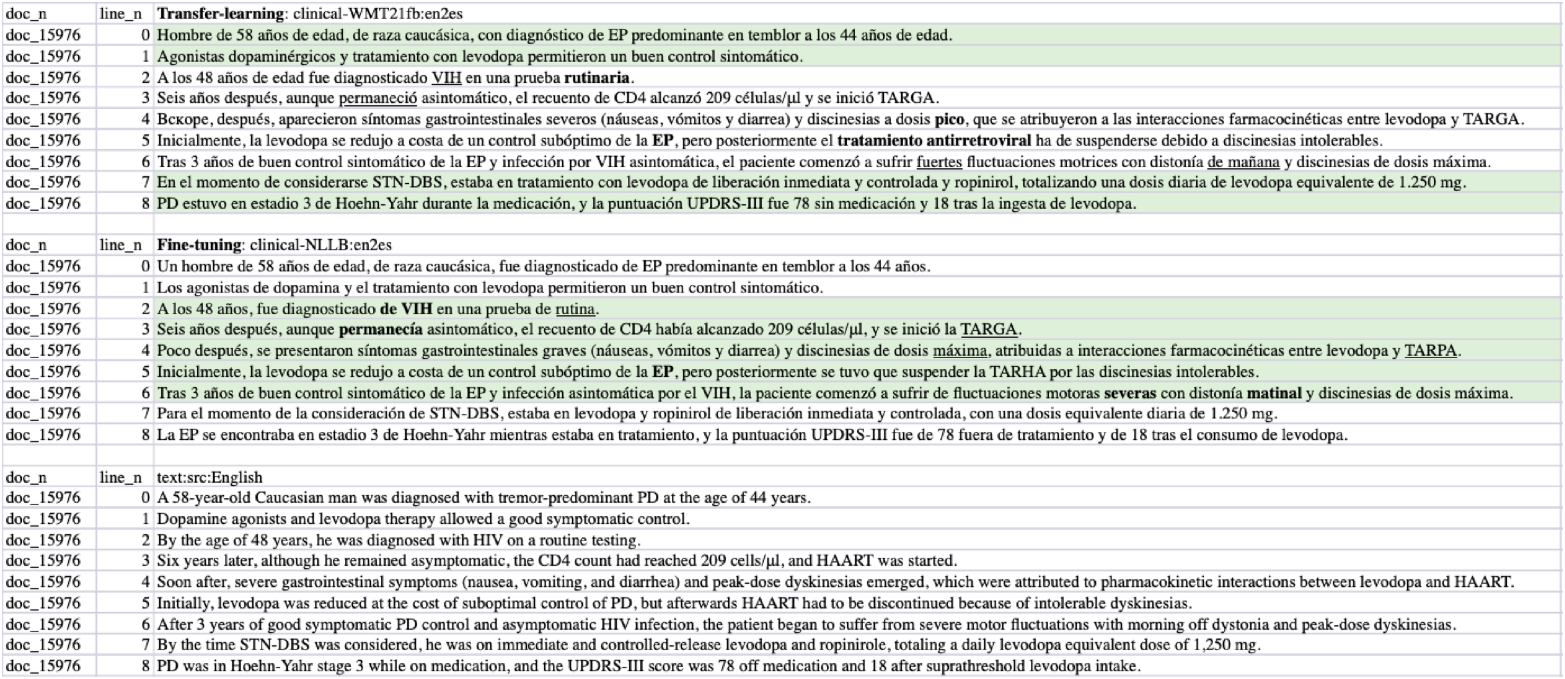
Task-1 Cases/Sentences EN-ES Translation Examples: clinic-WMT21fb *vs* clinic-NLLB.

**Figure 10:**
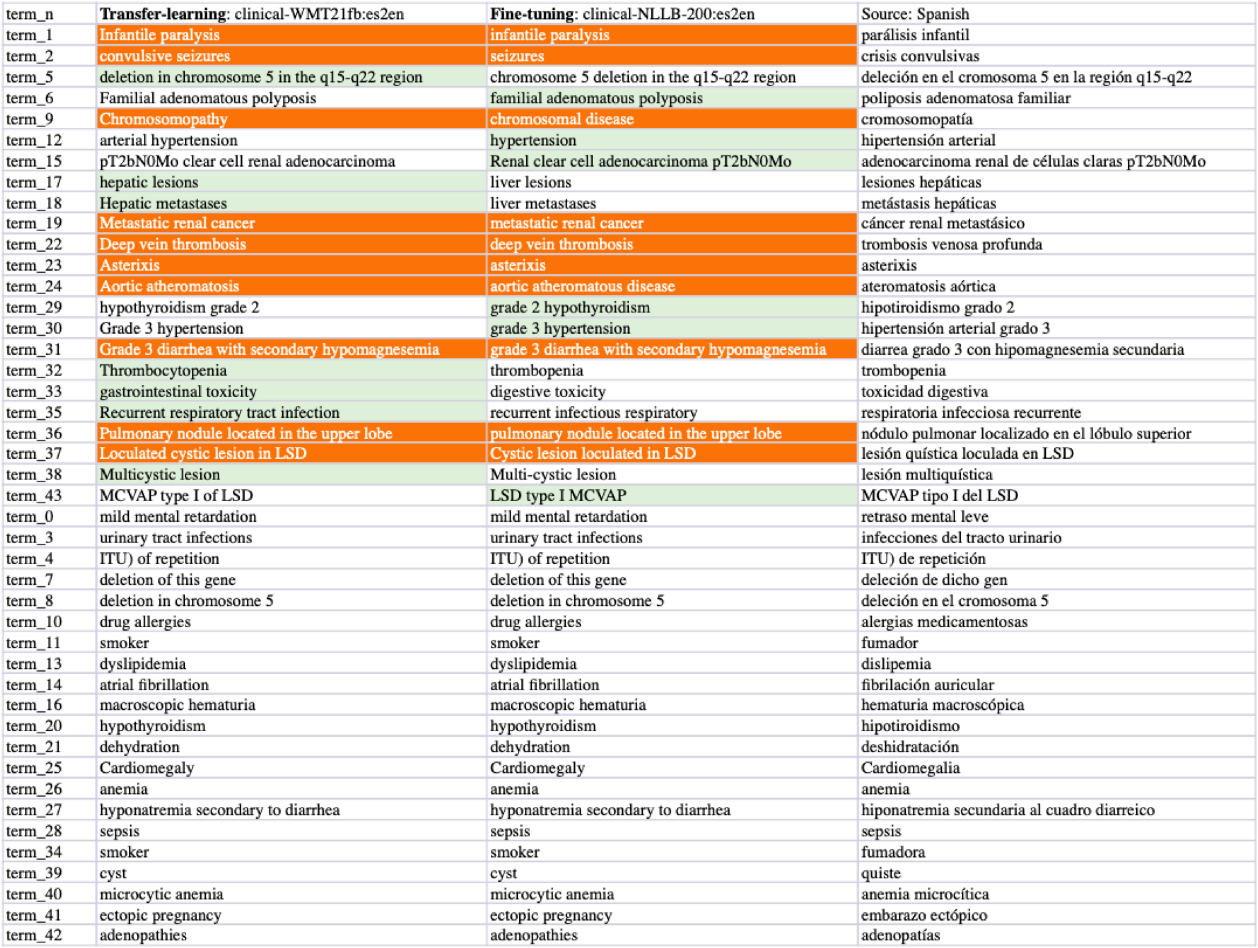
Task-2 Clinical Term ES-EN Translation Examples: clinic-WMT21fb *vs* clinic-NLLB.

**Figure 11:**
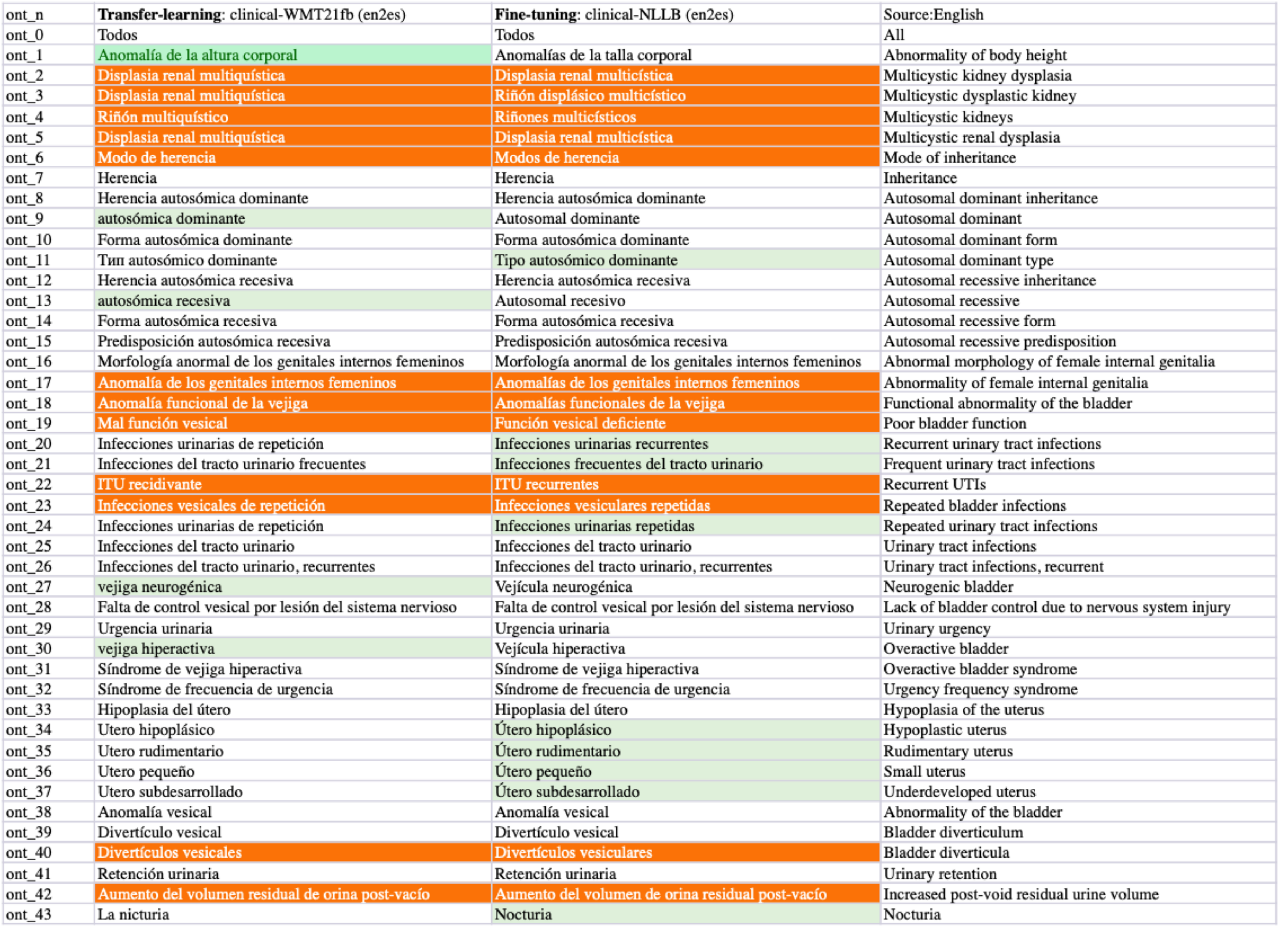
Task-3 Concept EN-ES Translation Examples: clinic-WMT21fb *vs* clinic-NLLB.

We list sampled error analyses on the outputs from the fine-tuned WMT21fb and NLLB models in Figures 9–11 for the three tasks on translations of sentences, terms, and concepts. The preferred translations are highlighted in green colour and “both sounds ok” is marked in orange.

From the comparisons of sampled output sentences, we discovered that the most frequent errors in a fine-grained analysis include *literal* translations, *oral vs written* languages, translation *inconsistency*, *inaccuracy* of terms, *hallucination/made-up* words, and *gender*-related errors such as feminine vs masculine, in addition to the standard fluency and adequacy that have been commonly used by traditional MT researchers (64). For instance, in Figure 9, the first two sentences (line 0 and 1) from the clinical-WMT21fb model are more written Spanish than the clinical-NLLB model which outputs are more oral Spanish. However, line 6 from the clinical-WMT21fb model includes the words “fuertes” which means “strong” that is not as accurate as “severas/severe” from the other model. In addition, “de manana” in the same line is less natural than “matinal” from clinical-NLLB. Regarding gender-related issues, we can see the examples also in line 6, where clinical-WMT21fb produced “el paciente” in masculine while clinical-NLLB produced “la paciente” in feminine. However, the source did not say what gender is “the patient”. Regarding literal translation examples, we can see in Figure 11, line ont-19 shows that clinical-WMT21fb gives more literal translation “Mal función vesical” than the preferred one “Función vesical deficiente” by clinical-NLLB when translating “Poor bladder function”. The neural model output hallucinations can also be found in Figure 11, e.g. “Vejícula” does not exist and it is like a mix of “vejiga” and “vesicula” in Line ont_27; similarly, in Line ont_2, “multicística” is a mix of Spanish and English, because the correct Spanish shall be “multiquística”.

As we mentioned in Section 4., there are 4% Russian tokens in the English-to-Spanish translation outputs from the Clinical-WMT21fb model which can be observed in Figures 9 and 11. However, they are meaningful tokens instead of nonsense, e.g. the Russian tokens in Figure 9 from line_n 4 means “soon” and in Figure 11 means “type of” from ont_11.

## 6 Discussions and conclusions

To boost the knowledge transformation for digital healthcare and make the most knowledge out of available clinical resources, we explored the state-of-the-art neural language models regarding their performances in clinical machine translation. We investigated a smaller-sized multilingual pre-trained language model (s-MPLM) Marian from the Helsinki NLP group, in comparison to two extra-large MPLM (xL-MPLM) NLLB and WMT21fb from Meta-AI. We also investigated the transfer-learning possibility in clinical domain translation using xL-MPLM WMT21fb. We carried out data cleaning and fine-tuning in the clinical domain. We evaluated our work using both automatic evaluation metrics and human expert-based evaluation using the HOPE (63) framework.

The experiment also leads to very far-reaching conclusions about MT models and their design, test, and applications:

(1) The bigger model does not mean that the quality is better. This premise proved to be false, evidently because researchers need vast amounts of data to train very large models and very often such data is not clear enough. On the contrary, when we clean the data very well for fine-tuning, we can bring the model quality much higher in specific domains, e.g. clinical text. We reached the point when the data quality was more important than the model’s size.
  One key takeaway for researchers and practitioners from this point is that if it is possible to get 250,000 clean segments in a new low-resource language, it is capable of fine-tuning large language models (LLMs) and get a good enough engine in this language. Then, the next step is to continue to get clean data by post-editing translation output from that engine. This is a very important conclusion for “low resource languages”.
(2) Automated metrics deliver an illusion of measurement – they are a good tool for iterative stochastic gradient descent during training, but they do not measure quality (only some sort of similarity), are not compatible when any of the underlying factors change, provide results on a non-uniform scale even on their interval of validity, in general are not sufficiently reliable, and may be misleading. We can’t rely on automatic metrics alone. Instead, human translation quality validation is a must and such validation can deny and reverse the results of automatic measurement.

## Data Availability

The original contributions presented in the study are included in the article/Supplementary Material, further inquiries can be directed to the corresponding authors.

## Footnotes

^1^ https://codalab.lisn.upsaclay.fr/competitions/6696

^2^This paper reports systematic investigation findings based upon the preliminary work from (28–30)

^3^ https://huggingface.co/Helsinki-NLP

^4^ https://github.com/facebookresearch/fairseq/tree/main/examples/wmt21

^5^ https://temu.bsc.es/clinspen/

^6^The 100 examples for evaluators were randomly selected from the test dataset.
